# Whole-Genome Sequence Analysis Reveals the Origin of the Chakouyi Horse

**DOI:** 10.3390/genes13122411

**Published:** 2022-12-19

**Authors:** Ying Li, Yu Liu, Min Wang, Xiaoran Lin, Yuanyuan Li, Tao Yang, Mo Feng, Yao Ling, Chunjiang Zhao

**Affiliations:** 1 Equine Center, China Agricultural University, Beijing 100193, China; 2 College of Animal Science and Technology, China Agricultural University, Beijing 100193, China; 3 National Engineering Laboratory for Animal Breeding, Beijing 100193, China; 4 Key Laboratory of Animal Genetics, Breeding and Reproduction, Ministry of Agriculture, Beijing 100193, China; 5 Beijing Key Laboratory of Animal Genetic Improvement, Beijing 100193, China

**Keywords:** Chakouyi horse, population structure, origin, DMRT3, the ability to pace, selection signature

## Abstract

The Chakouyi horse is an ancient Chinese indigenous horse breed distributed in Gansu Province in northwestern China, and is also one of the key breeds protected by the government. However, the origin of the Chakouyi horse remains unclear. As it is distributed in a key region of the Silk Road, it was speculated that the origin of the Chakouyi horse might involve the foreign horse breeds found along this ancient commercial artery. In this study, whole-genome resequencing data of 12 horse breeds, including both indigenous and foreign horses, were applied to reveal the genetic relationships between the Chakouyi horse and other breeds, as well as the ancestry of this ancient breed. An analysis of the population structure and admixture showed that there is no close genetic affinity between the Chakouyi horse and the foreign horses while Chinese indigenous horse populations were grouped together in accordance with their geographic locations, and the Chakouyi horse showed a closer relationship with Kazak horses, Mongolian horses, and Tibetan horses. The results from the ancestral composition prediction indicated that the Kazak horse and the Mongolian horse might be two ancestors of the Chakouyi horse. Furthermore, the genome-wide selection signature analysis revealed that the DMRT3 gene was positively selected in the Chakouyi horse and related to the gait trait of the breed. Our results provide insights into the native origin of the Chakouyi horse and indicate that Kazak and Mongolian horses played important roles in the formation of the Chakouyi horse. Genetic communication between the Chakouyi horse and other horse populations could be attributed, at least partially, to population migrations and trade activities along the ancient commercial routes.

## 1. Introduction

The Chakouyi horse is an indigenous horse breed distributed in the alpine areas of Tianzhu County, Gansu Province, in northwestern China. The Chakouyi horse is a hardy local breed with a wither height of approximately 130 cm, and it is famous for its inborn ability to pace. Chakouyi horses have a history over 2000 years, and were excellent post horses in ancient times and were also used for military purposes or served as agricultural work animals [1]. In recent decades, the popularization of mechanization in agriculture and the modernization of transportation have caused a significant decrease in the population of Chakouyi horses. The Chakouyi horse has been listed as a key breed to be protected by the Chinese government since 2006. Currently, the utilities of Chakouyi horses have shifted from draft horses to sport horses, and the local people have more motivation to breed them; thus, the number of horses has increased slightly, but more preservation efforts are still needed. 

Genetic studies on the Chakouyi horse are insufficient. Previous studies on Chinese indigenous horses with mtDNA showed that the haplotype diversity of Chakouyi horses was relatively high compared with other Chinese indigenous horse breeds (CHBs), which indicated that there are plentiful maternal lines in Chakouyi horses [2]. Analysis with Y-chromosomal microsatellite DNA revealed that the Chakouyi horse has a close genetic link with the Mongolian horse [3]. Another study with whole genome SNP array indicated that the Chakouyi horse is one of the most likely ancestors of the Jinjiang horse, an indigenous horse breed from the southeastern coasts of China [4]. The Chakouyi horse originated in a crucial section of the Silk Road, and it has been hypothesized that Chakouyi horses might have genetic links with the foreign horse populations along this ancient commercial route [5]. However, the origin of the Chakouyi horse remains unclear.

In recent years, whole genome resequencing data have been widely used in evolutionary studies, which provides a significantly higher resolution among populations than the data derived from mtDNA or the limited loci of the genome, and could reveal in-depth genetic information of the studied populations. To verify the hypothesis that the formation of the Chakouyi horse is related to the horse breeds along the ancient Silk Road, its genetic relationships with foreign and indigenous horse populations were analyzed with genomic data in the present study, and the ancestral composition of the Chakouyi horse was estimated and the selection signatures in Chakouyi horses were detected. The findings of the present study provide insight into the genetic background and origin of the Chakouyi horse, and will facilitate efforts to conserve the ancient breed. 

## 2. Materials and Methods

### 2.1. Sample Collection and Datasets

Blood samples of 85 Chinese indigenous horses were collected, including 35 Chakouyi horses from Tianzhu County of Gansu Province, 25 Kazak horses from Yili District of Xinjiang Province, and 25 Baise horses from Baise County of Guangxi Province. Genomic DNA was extracted using a rapid blood genomic DNA extraction kit (Tiangen Technology Co., Ltd., Beijing, China) and sequenced using the Illumina HiSeq X platform with 5× depth on average. In addition, the whole-genome resequencing data of 88 horses from 3 CHBs, 5 foreign horse breeds, and 4 Przewalski’s horses were downloaded from the European Nucleotide Archive (https://www.ebi.ac.uk, accessed on 18 October 2020). The three CHBs consisted of 24 Tibetan horses from Tibet, 17 Debao ponies from Guangxi, and 15 Mongolian horses from Inner Mongolia, while the 5 foreign horse breeds were Arabian horses (*n* = 7), Thoroughbreds (*n* = 8), Hanoverian horses (*n* = 4), Holsteiner horses (*n* = 5), and Akhal-Teke horses (*n* = 4). A total of 173 horses were included in the subsequent analysis. The details of the downloaded horse data are shown in Table S1. All of the sampling work involved in this study was conducted according to the regulations approved by the ethical committee of the China Agricultural University.

To analyze the genetic relationships between the Chakouyi horse and the foreign horse breeds and other CHBs, two datasets were established: (1) All of the foreign horse breeds, the Chakouyi horses and the Przewalski’s horses (outgroup) were merged into a Chakouyi-foreign horse dataset (*n* = 67), and (2) the Chakouyi horses, the other CHBs and the Przewalski’s horses (outgroup) formed a Chakouyi-Chinese horse dataset (*n* = 145).

### 2.2. SNP Calling

The reference genome sequence for the domestic horse, EquCab 3.0, was downloaded from Ensembl (version 101) (ftp://ftp.ensembl.org/pub/release-101/fasta/equus_caballus/DNA/, accessed on 10 December 2020). The clean reads were aligned to the reference genome using BWA (version 0.7.17) with default parameters [6]. Multiple alignment and duplicate reads were removed using SAMtools (1.10) [7] and the Genome Analysis Toolkit (4.1.7.0) [8]. Variant calling was performed using HaplotypeCaller with the options “stand emit conf 10” and “stand call conf 30” to detect insertion/deletions (Indels) and SNPs. SNPs were separated through the “selectVariant” option in the Genome Analysis Toolkit and the sex chromosomes were discarded. 

SNPs were filtered using PLINK 1.9 (Purcell, 2007) [9], and the following SNPs were discarded: (1) SNPs with Hardy–Weinberg equilibrium *p*-value < 1 × 10^−5^; (2) SNPs that were missing more than 10% of their genotype data; and (3) SNPs with a minor allele frequency < 1%. Individuals with more than 10% missing genotyped data were also removed. After filtering, the total number of SNPs left in the Chakouyi-foreign horse dataset was 22,050,722, while there were 26,355,741 filtered SNPs in the Chakouyi-Chinese horse dataset. All of the filtered SNPs were annotated using SnpEff [10]. 

### 2.3. Population Divergence

All of the SNPs were pruned using PLINK1.90 with a window size of 100 variants, a step size of 50, and a pairwise r^2^ threshold of 0.2 (Indep-pairwise 100 50 0.2). To further explore the genetic structure of the Chakouyi horse and the other five foreign horse breeds, the neighbor-joining (NJ) tree was constructed using MEGA v6 based on the distance matrix, and displayed by FigTree v1.4.0. Principal component analysis (PCA) was conducted with GCTA 1.92 software [11]. The genetic relationship matrix and the covariance matrix were inferred from the PLINK format files (.ped and .map) with the parameters “-make-grm-pca 3”. The PCA biplot was plotted with ggplot2 (R Packages). ADMIXTURE 1.3.0 software [12] was applied to cluster the samples and to evaluate the genetic structure in the dataset, and the number of clusters (K) was set from 2 to 6.

To investigate the relationship between the Chakouyi horse and the other CHBs, a phylogenetic tree, PCA, and analysis of the shared ancestry were also constructed or conducted with the Chakouyi-Chinese horse dataset using the same methods mentioned above.

### 2.4. LD Decay and Genetic Diversity

Linkage disequilibrium (LD) levels for the CHBs were assessed by the genotype correlation coefficient (r^2^) between any two loci (within and between different chromosomes). The software PopLDdecay [13] was applied for the LD analysis, and visualization of LD decays of the horse populations across the whole genome was generated using R scripts. Based on the autosomal SNP data, the genetic diversity indexes of the studied populations were calculated. The homozygosity and inbreeding coefficient of individuals were computed with the -het command of Plink software, and the observed heterozygosity (Ho) and expected heterozygosity (He) were calculated with the -hardy command.

### 2.5. Detection of Migrations

Treemix v1.12 [14] software was used to clarify the historical migration and splits between the Chakouyi horse and other CHBs, and a migration event analysis was conducted at the population level. The f index indicating the fraction of the variance in the covariance matrix of the samples calculated by the model covariance matrix was applied to identify the number of modeled migration event that best fit the data. After converting the PLINK format SNP matrix to Treemix format with the software plink2treemix.py, the ML tree was constructed with the Chakouyi-Chinese horse dataset.

### 2.6. Identical by Descent Analyses

The genome-wide SNP data of domestic horse individuals of the Chakouyi-Chinese horse dataset served as the input for identical by descent (IBD) detection. The frequencies of shared haplotypes between the Chakouyi horse and each of the other CHBs were estimated per 10,000 bp bins using IBDLD (v3.37). The parameters were set as “-plinkbf int evolution-method GIBDLD–ploci 10-nthreads 24-step 0-hiddenstates 3-segment–length 10-min 0.8”. The calculation of the normalized IBD (nIBD) between the Chakouyi horse and each of the other CHBs was conducted as follows: nIBD = cIBD/tIBD, where cIBD is the count of all haplotypes IBD between the Chakouyi horse and each of the other CHBs, and tIBD indicates the total pairwise comparisons between the Chakouyi horse and each of the other CHBs.

### 2.7. Formal Test of Ancestor Admixture

The most likely ancestry of Chakouyi horses was estimated using the f4 ratio estimation method of the ADMIXTOOLS Software Package with default parameters. The f4 ratio was calculated with f4 (A, O; X, C)/f4 (A, O; B, C), in which population X is an admixture of populations B and C. In this study, the Debao pony was set as A, the Kazak horse as B, the other CHBs as C, the Chakouyi horse as X, and Przewalski’s horses as O.

### 2.8. Selective Signatures in Chakouyi Horses

To detect the genomic regions related to selection in the Chakouyi horses, we calculated the population differentiation statistic (F_ST_). The F_ST_ between the Chakouyi horse and the other horses was quantified using a sliding 20-kb window with a 5-kb step by vcftools-0.1.16 [15]. After the analysis, all of the genes in the top 1% of the regions with significantly high F_ST_ values were annotated to the horse reference genome.

### 2.9. SNP Validation of the DMRT3 Gene

The primers and PCR-RFLP method for genotyping the mutation associated with gait traits described previously [16] were used in the present study to investigate the alleles related to the ability to pace in Chakouyi horses. For further confirmation, the PCR products were sent to BGI (Beijing, China) for sequencing.

## 3. Results

### 3.1. Genetic Relationship between the Chakouyi Horse and Foreign Horse Breeds

**Figure 1:**
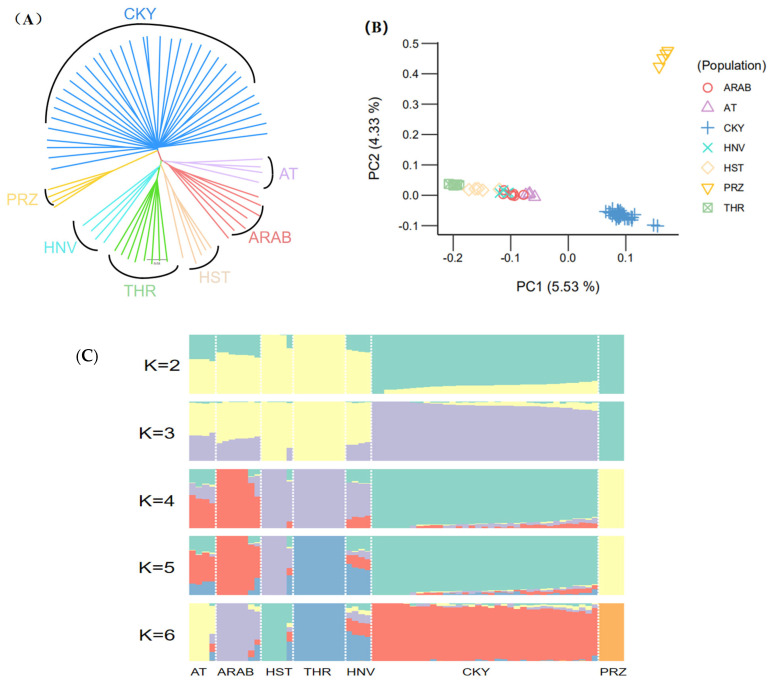
Population genetic relationship between the Chakouyi horse and foreign horse breeds. (**A**) Neighbor-joining tree of the Chakouyi horses and the foreign horses constructed with the genome-resequencing data. The populations studied are indicated with different colors and the names of the breeds. (**B**) PCA results of the Chakouyi horse and foreign horse breeds. The studied horse populations are indicated with different colors. (**C**) ADMIXTURE analysis of the Chakouyi horse and the foreign horse populations. Each column indicates an individual, and each column group represents a horse population. AT, Akhal-Teke horse; Arab, Arabian horse; HST, Holsteiner horse; THR, Thoroughbreds; HNV, Hanoverian horse; CKY, Chakouyi horse; PRZ, Przewalski’s horse.

Using the Chakouyi-foreign horse dataset, we explored the relationship among the studied populations to identify the horse breeds closely related to the Chakouyi horse. The neighbor-joining tree showed that the Chakouyi horses and the foreign horses were classified into two clusters (Figure 1A). Additionally, the Przewalski’s horses were clustered separately and had a closer genetic link with the Chakouyi horse. The foreign horse breeds were split into two geographically structured clades. The Hanoverian horses and Holsteiner horses are closely related to the Thoroughbreds, while the Akhal-Teke horses and Arabian horses are clustered together, which is in accordance with the geographical locations and breeding history of these foreign horse breeds. The results above show that there was no close genetic relationship between the Chakouyi horse and any of the foreign horse breeds. The PCA results are consistent with the neighbor-joining tree results, which also showed that the Chakouyi horse is separate from the foreign horses studied (Figure 1B and Figure S1).

Based on the genetic co-ancestry analyses [12], we assigned all individuals into known groups by varying the number of presumed ancestral populations (K ranging from 2 to 6). The ADMIXTURE results showed clear differences between the Chakouyi horse and the foreign breeds, while the foreign Asian breeds (Akhal-Teke horses and Arabian horses) and the European horse breeds (Hanoverian horses, Holsteiner horses, and Thoroughbreds) showed similar ancestral compositions (Figure 1C). These results are in accordance with those from the phylogenetic analysis and the PCA.

### 3.2. Genetic Relationships between the Chakouyi Horse and Other Chinese Horse Breeds

**Figure 2:**
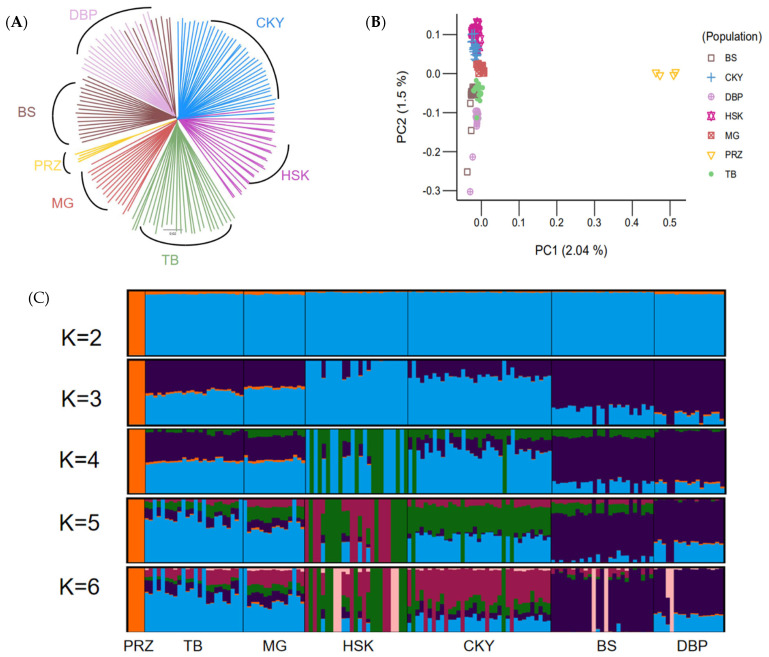
Population genetic relationship between Chakouyi horses and the other Chinese horse breeds. (**A**) Relationships among the six studied CHBs illustrated by a neighbor-joining tree. The populations studied are indicated with different colors and the names of the breeds. (**B**) PCA plot of the CHBs. The studied populations are indicated with different colors. (**C**) ADMIXTURE analysis of the Chakouyi horse and other CHBs. Each column indicates an individual, and each column group represents a horse population. CKY, Chakouyi horse; HSK, Kazak horse; MG, Mongolian horse; TB, Tibetan horse; BS, Baise horse; DBP, Debao pony; PRZ, Przewalski’s horse.

A neighbor-joining (NJ) tree was constructed with the Chakouyi-Chinese horse dataset. The results of the phylogenetic tree in Figure 2A show that Przewalski’s horses and CHBs were divided into two clusters. Among the horse populations in China, the two breeds from South China, the Baise horse and the Debao pony, were clustered together, while the Chakouyi horse was clustered with the Mongolian horse, the Kazak horse, and the Tibetan horses. This indicates that the Chakouyi horse has close genetic links with them. Among the four breeds, there was a closer genetic relationship between the Chakouyi horse and the Kazak horse. The result of the PCA conducted with the Chakouyi-Chinese horse dataset (Figure 2B and Figure S2) was consistent with those of the phylogenetic analysis. The admixture analysis detected admixture patterns in accordance with the results from the phylogenetic tree and the PCA. The Baise horse and the Debao pony shared a similar genetic background, while there was admixture between the Chakouyi horse and the other three CHBs, especially an evident admixture between the Chakouyi horse and the Kazak horse (Figure 2C).

**Figure 3:**
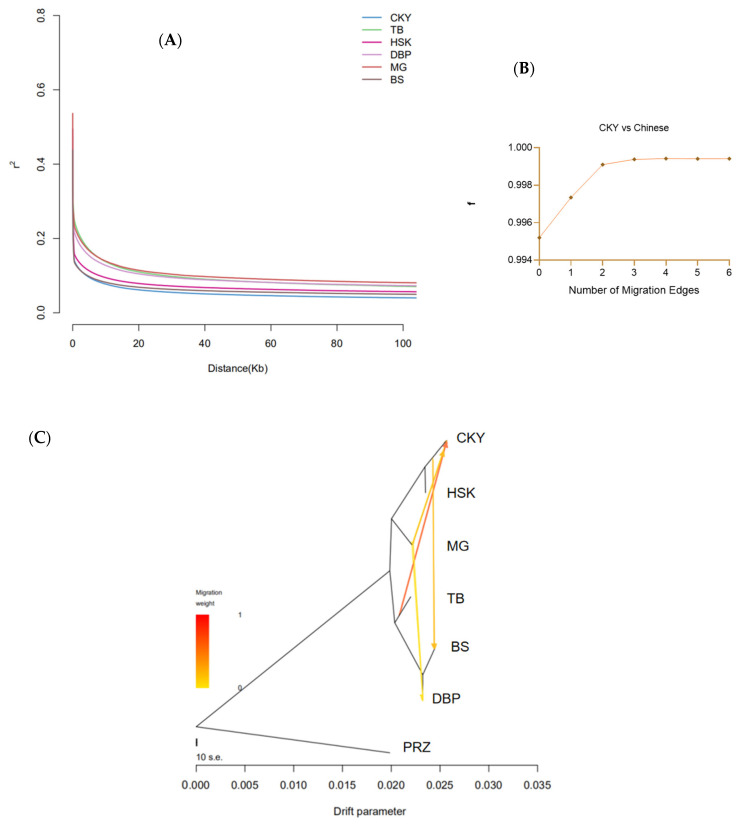
Linkage disequilibrium decay and migration detection of the studied CHBs. (**A**) Linkage disequilibrium decay of the studied CHBs. The studied populations are indicated with different colors. (**B**) Modeling the number of modeled migration events. More migration edges did not further increase the variance explained by the phylogenetic model, as the f index reached an asymptote above 4 migration edges, so m = 4 was chosen as optimum migration mode in the dataset. (**C**) Migrations detected among the studied CHBs by the TreeMix program with four migration events. CKY, Chakouyi horse; HSK, Kazak horse; MG, Mongolian horse; TB, Tibetan horse; BS, Baise horse; DBP, Debao pony; PRZ, Przewalski’s horse.

The linkage disequilibrium (LD) coefficient of each CHB was calculated (Figure 3A). The Chakouyi horses showed the fastest LD decay rate and the smallest LD decay distance, while the Mongolian horses, Debao ponies, and Tibetan horses had a relatively slow LD decay rate and large LD decay distances. However, the genetic diversity revealed with the heterozygosity in the Chakouyi horses at a genomic level was relatively low (Table S2). Four migration events among the studied CHBs were detected with the TreeMix program as m = 4 (Figure 3B,C). The admixture proportions of the migration events from the Tibetan horses to the Chakouyi horses, and the Chakouyi horses to the Baise horses were relatively high. The other two migration events from the Mongolian horses to the Chakouyi horses and from Mongolian horses to Debao ponies were also detected, but they showed relatively low admixture proportions. The migration model mainly showed the gene flows within northern horses and those from northern horses to southwestern horses.

### 3.3. Identical by Descent Analyses of the Chakouyi Horse

**Figure 4:**
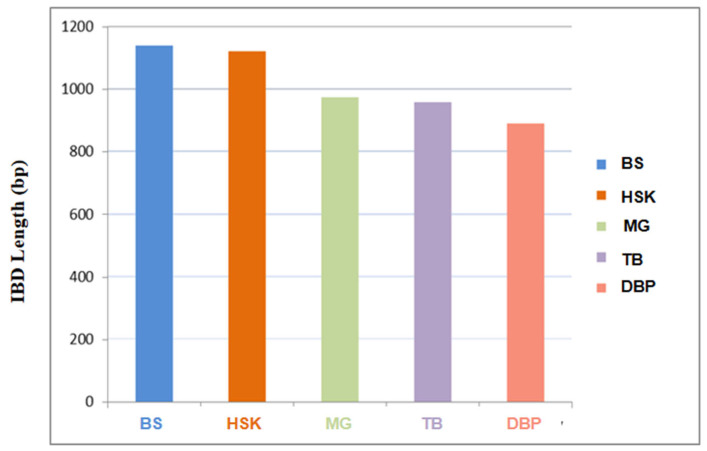
The estimation of identity by descent shared between the Chakouyi horse and other CHBs. HSK, Kazak horse; MG, Mongolian horse; TB, Tibetan horse; BS, Baise horse; DBP, Debao pony.

The results of the IBD analysis revealed that Chakouyi horses are closely related to Baise horses and Kazak horses. In addition, the Baise horse had a significantly higher shared IBD with the Chakouyi horse compared with the other CHBs, serving as additional evidence of migration from the Chakouyi group to the Baise group. The Debao pony showed the shortest IBD segment length, shared with the Chakouyi horse, indicating a distant genetic relationship between them (Figure 4).

### 3.4. Estimation of Possible Ancestry with a Formal Test of ADMIXTURE

The above analyses indicate that the Chakouyi horse has a diverse Chinese native origin, but the possible ancestry of the Chakouyi horse remains unknown. Therefore, the f4 ratio estimation algorithm (f4 ratio = f4 (A, O; X, C)/f4 (A, O; B, C)) from the ADMIXTOOLS Software Package was used to conduct a further analysis. The Chakouyi horse is the target population (X) and the Kazak group (C) is the most likely ancestor of the Chakouyi horse in the estimation of the f4 ratio. In the output of the analysis, the results are positive only when the value of the f4 ratio is above zero, and the greater the f4 ratio, the more likely it is that the B breed in the algorithm and the Kazak horses are the ancestors of the Chakouyi horses. The results show that the Kazak horses and the Mongolian horses are the most likely ancestors of the Chakouyi horses (Table 1).

### 3.5. Detection for Signatures of Selection

**Figure 5:**
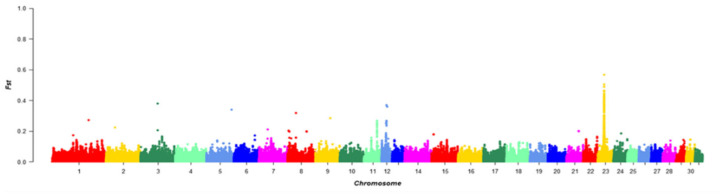
Manhattan plot of the F_ST_ values between the Chakouyi horses and other horses worldwide. The F_ST_ values are calculated for each 20 kb autosomal window. Adjacent chromosomes were indicated with different colors to make a better distinction of their borders.

F_ST_ was calculated for each SNP between the Chakouyi horse and the other horse breeds. Annotation was carried out for the genes in the top 1% of the F_ST_, and 488 genes were identified. The strongest selection was detected on ECA23 (*Equus caballus* autosome 23) between 22,385,001 and 22,405,000 bp of the chromosome. Notably, the DMRT3 gene was located in the screened region, which has previously been reported to have a predominant effect on the gaiting ability in Icelandic horses (Figure 5) [17].

### 3.6. Genotyping of the DMRT3 Gene

The PCR products of the DMRT3 gene of the Chakouyi horses were sequenced, and the known ECA23:g.22999655C > A mutation reported by previous studies was also identified in the Chakouyi horses. The frequencies of the genotypes and alleles at the locus in the Chakouyi horses were investigated with PCR-RFLP (Figure S3). The genotyping results of the Chakouyi horses and the other reported horse breeds are shown in Table 2. The Chakouyi horse had the second highest frequency of the AA genotype (0.9250) or A allele (0.9625) among the studied breeds (Table 2), only lower than that of the Tennessee Walker, and the A allele was related to the ability to pace, as reported in a previous study [17].

## 4. Discussion

### 4.1. The Chakouyi Horse and the Hexi Corridor

The Chakouyi horses are mainly distributed in Tianzhu County of Wuwei City in Gansu Province. Wuwei City is a key part of the Hexi Corridor, which is the most important passage from Xi’an to Xinjiang and Central Asia, and has also been a traditional horse-raising area since the Han Dynasty (B.C.202–A.D.220) [1]. The Chakouyi horse was used as a post horse in the ancient Chakouyi Station and thus was named for the prefecture of the place [1]. As the Hexi Corridor is part of the Silk Road [20], and horses played an important role in commercial activities in ancient times, it is necessary to investigate the genetic relationships between the Chakouyi horse and horse breeds distributed in other areas along the Silk Road. In the present study, horses from the regions related to the ancient road, such as the Kazak horse, Arabian horse, Akhal-Teke horse, and some European horses, were included for the analysis at the genomic level.

### 4.2. The Genetic Links between the Chakouyi Horse and the Foreign Breeds

The results of the phylogenetic tree and the PCA showed that the Chakouyi horse is clearly separate from the five foreign horse breeds, including the Arabian horses, Akhal-Teke horses, Holsteiner horses, Hanoverian horses, and Thoroughbreds, while the foreign breeds were clustered together. There is a remarkable difference in genetic background between the Chakouyi horse and the foreign breeds revealed in the analysis of their population structure. These results indicated that there is no close genetic relationship between the Chakouyi horse and foreign breeds, including the Arabian and Akhal-Teke horses, which were distributed along the ancient Silk Road and were found relatively near China. Overall, the Chakouyi horse does not show close genetic links with the studied foreign breeds, and this phenomenon seems to follow the isolation by distance patterns due to the geographical distribution of the breeds, which was similar to another study on the Jinjiang horse [4]. In the present study, only limited genomic data of foreign breeds could be retrieved from public databases and applied in the analyses. Although the studied CHBs were sampled in their original places, it was not guaranteed that all of them were purebreds as there were no detailed pedigree records for the CHBs. These may cause a possible bias in the analyses. Therefore, additional studies with larger sample sizes and known pedigree information are still needed to verify the outcomes.

### 4.3. Native Origin of the Chakouyi Horse

As the Chakouyi horse did not show a close genetic relationship with foreign horses, we further investigated its genetic links with other CHBs. The five other CHBs, including the Kazak horse, the Mongolian horse, the Tibetan horse, the Baise horse, and the Debao pony, representing the horse populations of the main horse-raising areas of China, were applied in this study. The results of the phylogenetic tree, PCA, and admixture analysis indicated that the Chakouyi horse has close genetic relationship with the Kazak horse, the Mongolian horse, and the Tibetan horse, which is in line with the results revealed by Y-chromosomal markers and mtDNA [2,3], while the Baise horse and the Debao pony have close genetic links. The migration detection, IBD analysis, and estimation of possible ancestry further revealed that the formation of the Chakouyi horse was closely related to the Kazak horse, the Mongolian horse, and the Tibetan horse. The Kazak horses and Mongolian horses are the most likely ancestors of the Chakouyi horses, which is in accordance with the known history of the Chakouyi horse.

The Mongolian horse has long been the most popular indigenous horse breed in China, and it is mainly distributed in Inner-Mongolia Province, which is to the north of Gansu Province. As two main adjacent horse-raising areas, there were frequent gene exchanges between the two breeds [21]. For centuries, Tibetan people have lived in Tianzhu County where the Chakouyi horse is mainly distributed [1]. They used to donate their high-quality horses to the local temples. Tibetan horses might have been introduced into Tianzhu County by the Tibetan people. In the Ming Dynasty (A.D.1368–1644), a market for exchanging tea for horses was established in Tianzhu County, and horses from Inner-Mongolia and the Qinghai–Tibet Plateau were gathered in the place, which facilitated gene exchange between the introduced horses and the local horses [22]. Our previous study with Y chromosomal microsatellites also indicated that the Mongolian horse, the Chakouyi horse, and the Tibetan horse have close genetic links [3]. The Kazak horse is an ancient indigenous breed distributed in Xinjiang Province along the Silk Road. There are records of the introduction of Kazak horses from Xinjiang Province to Xi’an through the Hexi Corridor [23], which led to a gene exchange between the Kazak horses and horse populations in the Hexi Corridor.

Migration events from the breeds of northern China to those of southern China, such as gene flow from the Chakouyi horse to the Baise horse, and from the Mongolian horse to the Debao pony, were also detected. There is the Tibetan-Yi Corridor along the north-south oriented rivers and valleys in the boundary between the southwestern and northwestern provinces of China, which served as a crucial route for ancient people migrating from the northwestern provinces to Southwestern China. The corridor has many connections with the ancient Tea Horse Road, and it eventually provided a route for gene flow between horse populations in northern and southern China [4]. On the whole, the results proved that there were gene flows along the Silk Road and the Hexi Corridor. Therefore, the close relationships between the Chakouyi horse and the other CHBs could be attributed to their adjacent distribution areas and migrations along the ancient commercial routes.

### 4.4. Selective Signature at the Genomic Level of the Chakouyi Horse

The Chakouyi horse was used as a post horse in ancient times, and a high proportion of individuals in the breed have an inborn ability to pace [1], which is a desirable gait for long-distance riding due to it being less bumpy than trotting. Currently, few CHBs possess the inborn ability to pace. It is likely that Chakouyi horses were subjected to selection by the local breeders for their pacing. The analysis of the selective signature at the genomic level revealed a significant selection signature at ECA23. The Chakouyi horse is famous for its inherent ability to pace, and the causative mutation of the trait was reported to be located in the DMRT3 gene at ECA23 in Icelandic horses [18], so we genotyped Chakouyi horses at the locus with a previously reported method [16] to check whether the reported locus also exists in Chakouyi horses. The results showed that the causative mutation is also harbored by the Chakouyi horses, and there is a high frequency of genotypes related to the ability to pace in Chakouyi horses. This result suggests that Chakouyi horses were selected for their ability to pace, and the same locus affected the trait in both Icelandic horses and Chakouyi horses.

### 4.5. The Conservation of the Chakouyi Horse

In the 1980s, there were over 30,000 Chakouyi horses in Gansu Province, most of which were used as agricultural work animals. Then, the population continuously decreased due to the mechanism of agriculture, and there were only approximately 5000 horses left in 2009 [24]. In recent years, Chakouyi horses have been increasingly used for riding and racing, and the government has carried out conservation plans to protect the breed, including preserving the horses’ inherent ability to pace. There are approximately 8000 Chakouyi horses in Gansu Province now. In the present study, the Chakouyi horses showed the smallest LD decay distance in the studied CHBs. This suggests that Chakouyi horses have not been subjected to intensive breeding activities, but the genetic diversity at the genomic level in the population is relatively low in the studied horse breeds. This suggests that more conservation efforts are required.

## 5. Conclusions

The Chakoukyi horse has no close links with foreign breeds, and it is more genetically related to other indigenous Chinese horse breeds. In particular, the Chinese breeds geographically near the Chakouyi horse and along the ancient commercial routes had strong genetic impacts on the formation of the Chakouyi horse. The breed’s inherent ability to pace should be given full consideration in the further utilization and breeding plans of the Chakouyi horse.

## Figures and Tables

**Table 1:** Estimation of the genome-wide possible ancestry of the Chakouyi horses *.

A	O	X	C	A	O	B	C	F4 Ratio	Std. err	Z (Null = 0)
THR	PRZ	CKY	HSK	THR	PRZ	MG	HSK	6.196961	6.520517	0.95 9d
THR	PRZ	CKY	HSK	THR	PRZ	BS	HSK	0.566136	0.145556	3.889 9d
THR	PRZ	CKY	HSK	THR	PRZ	TB	HSK	0.355764	0.088371	4.026 9d
THR	PRZ	CKY	HSK	THR	PRZ	DBP	HSK	0.329154	0.084602	3.891 9d
THR	PRZ	CKY	HSK	THR	PRZ	HNV	HSK	−0.03451	0.010505	−3.285 9d
THR	PRZ	CKY	HSK	THR	PRZ	HST	HSK	−0.03543	0.010896	−3.252 9d
THR	PRZ	CKY	HSK	THR	PRZ	ARAB	HSK	−0.03688	0.011185	−3.297 9d
THR	PRZ	CKY	HSK	THR	PRZ	AT	HSK	−0.05201	0.016226	−3.205 9d

* THR, Thoroughbred; PRZ, *Przewalski’s* horse; CKY, Chakouyi horse; HSK, Kazak horse; MG, Mongolian horse; BS, Baise horse; TB, Tibetan horse; DBP, Debao pony; HNV, Hanoverian horse; HST, Holsteiner horse; ARAB, Arabian horse; AT, Akhal-Teke horse.

**Table 2:** Genotype and allele frequencies of the DMRT3 gene mutation in Chinese indigenous horses and foreign horses.

Breed	Sample Number	Genotype Frequency			Allele Frequency		Resource
		CC	CA	AA	C	A	
Chaykouyi horse	40	0.0000 (0)	0.0750 (3)	0.9250 (37)	0.0375	0.9625	The present study
Datong horse	27	0.1482 (4)	0.6296 (17)	0.2222 (6)	0.4630	0.5370	Han et al. [18]
Kazak horse	17	0.3750 (6)	0.5000 (8)	0.1250 (2)	0.6250	0.3750	Han et al. [18]
Baise horse	33	1.0000 (33)	0.0000	0.0000	1.0000	0.0000	Han et al. [18]
Debao pony	40	1.0000 (40)	0.0000	0.0000	1.0000	0.0000	Han et al. [18]
Guizhou horse	23	1.0000 (23)	0.0000	0.0000	1.0000	0.0000	Han et al. [18]
Lichuan horse	18	1.0000 (18)	0.0000	0.0000	1.0000	0.0000	Han et al. [18]
Niqiang horse	46	0.9565 (44)	0.0435 (2)	0.0000	0.9783	0.0217	Han et al. [18]
Arabian horse	69	1.0000 (69)	0.0000 (0)	0.0000 (0)	1.0000	0.0000	Promerova et al. [19]
Akhal-Teke horse	43	1.0000 (43)	0.0000 (0)	0.0000 (0)	1.0000	0.0000	Promerova et al. [19]
TennesseeWalker	54	0.0000 (0)	0.0000 (0)	1.0000 (54)	0.0000	1.0000	Promerova et al. [19]
Icelandic horse	219	0.0411 (9)	0.4247 (93)	0.5342 (117)	0.2535	0.7465	Promerova et al. [19]

## Supplementary Materials

The following are available online at https://www.mdpi.com/article/10.3390/genes13122411/s1, Figure S1: PCA results of the Chakouyi horse and foreign horse breeds (PC3). Figure S2: PCA results of the Chakouyi horse and CHBs (PC3). Figure S3: Band patterns of PCR products of the DMRT3 gene digested with restriction enzyme *Dde I* on agarose gel. Table S1: Information on the downloaded data in the present study. Table S2: Statistics of diversity parameters of the studied Chinese and foreign horse populations at genomic level.
