# Supplementary material for: Whole-Genome Sequence Analysis Reveals the Origin of the Chakouyi Horse
Source: Genes (Basel). 2022 Dec 19;13(12):2411. doi: 10.3390/genes13122411 (PMC9778315; doi:10.3390/genes13122411)
Supplement: Supplementary file 1 [file genes-13-02411-s001.zip › genes-2037671-supplementary-proof(2022-12-18)/Figure S3.docx]

Figure S3 Band patterns of PCR products of the DMRT3 gene digested with restriction enzyme *Dde I* on agarose gel

Lane 1-2, CC genotypes; Lane 3-4, CA; Lane 5-9, AA; Lane 10, DNA standards.
