# Supplementary material for: Whole-Genome Sequence Analysis Reveals the Origin of the Chakouyi Horse
Source: Genes (Basel). 2022 Dec 19;13(12):2411. doi: 10.3390/genes13122411 (PMC9778315; doi:10.3390/genes13122411)
Supplement: Supplementary file 1 [file genes-13-02411-s001.zip › genes-2037671-supplementary-proof(2022-12-18)/Table S2.docx]

**Table S2**  Statistics of diversity parameters of the studied Chinese and foreign horse populations at genomic level*

| Population | N | F_genome_ | He | Ho | O (HOM) | E (HOM) |
| --- | --- | --- | --- | --- | --- | --- |
| CKY | 35 | 0.325246 | 0.275369 | 0.185225 | 2515219 | 2235829 |
| BS | 25 | 0.32816 | 0.273339 | 0.183072 | 2468948 | 2188440 |
| HSK | 25 | 0.311644 | 0.269921 | 0.185328 | 2440542 | 2179000 |
| TB | 24 | -0.010329 | 0.317785 | 0.321502 | 2260862 | 2273583 |
| MG | 15 | 0.125270667 | 0.289757 | 0.252622 | 2305597 | 2188000 |
| DBP | 17 | 0.065386471 | 0.300202 | 0.280239 | 2312284 | 2246412 |

*only the populations with sample size > 10 were calculated for the diversity parameters.

N：sample size

F_genome_：Inbreeding coefficient based on genome wide SNP calculation

He：Expected heterozygosity

Ho：Observed heterozygosity

O(HOM)：Average number of homozygotes observed

E(HOM)：Average number of expected homozygotes
