# Supplementary material for: Whole-Genome Sequence Analysis Reveals the Origin of the Chakouyi Horse
Source: Genes (Basel). 2022 Dec 19;13(12):2411. doi: 10.3390/genes13122411 (PMC9778315; doi:10.3390/genes13122411)
Supplement: Supplementary file 1 [file genes-13-02411-s001.zip › genes-2037671-supplementary-proof(2022-12-18)/Table S1.docx]

**Table S1** Information on the downloaded data in the present study

| Breed | Samples | Accession ID（NCBI） |
| --- | --- | --- |
| Tibetan horse | 24 | SRR8442553; SRR8442554; SRR8442555; SRR8442557; SRR8442558; SRR8442559; SRR8442562; SRR8442563; SRR8442564; SRR8442566; SRR8442567; SRR8442568; SRR8442569; SRR8442571; SRR8442572; SRR8442577; SRR8442578; SRR8443579; SRR8442580; SRR8442583; SRR8442584; SRR8442587; SRR8442599; SRR8442602 |
| Debao pony | 17 | SRR12072881; SRR12072882; SRR12072883; SRR12072884; SRR12072885; SRR12072886; SRR12072887; SRR12072888; SRR12072889; SRR12072890; SRR12072891; SRR12072892; SRR12072893; SRR12072894; SRR12072895; SRR12072896; SRR12072897; |
| Mongolian horse | 15 | SRR8442556; SRR8442560; SRR8442561; SRR8442570; SRR8442574; SRR8442576; SRR8442581; SRR8442582; SRR8442585; SRR8442591; SRR8442593; SRR8442594; SRR8442600; SRR8442601; SRR8442604; |
| Przewalski’s horse | 4 | SRR8442573; SRR8442575; SRR8442588; SRR8442589; |
| Arabian horse | 7 | ERR2179551; ERR1527951; ERR3465834; ERR3465835; ERR3465837; ERR3465839; ERR3465840 |
| Thoroughbred horse | 8 | SRR505867; SRR515205; SRR515211; SRR515202; SRR515212;  SRR515203; SRR515204; SRR515209 |
| Hannoverian horse | 4 | SRR2142163; SRR2142269; SRR1046151; SRR1046147; |
| Holsteiner horse | 5 | ERR2731058; ERR2731059; ERR2731060; ERR2731061; ERR2179547; |
| Akha-Teke horse | 4 | ERR1527947; ERR1527948; ERR1527949; ERR1527950; |
