# Supplementary figures and images for: Whole-Genome Sequence Analysis Reveals the Origin of the Chakouyi Horse
Source: Genes (Basel). 2022 Dec 19;13(12):2411. doi: 10.3390/genes13122411 (PMC9778315; doi:10.3390/genes13122411)

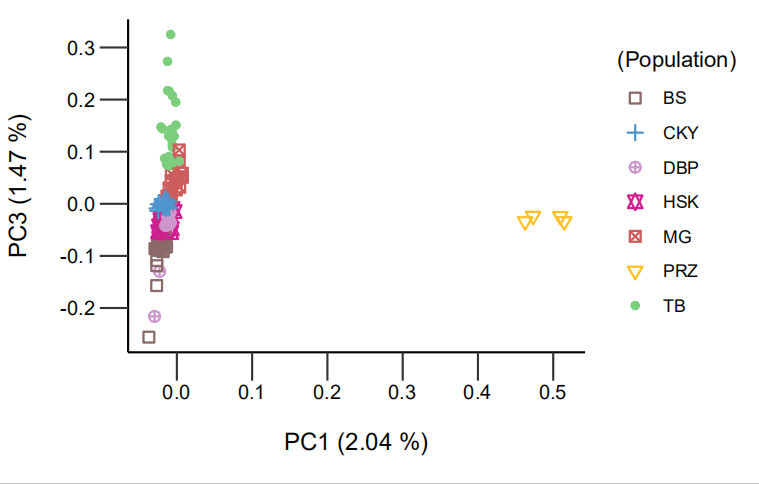

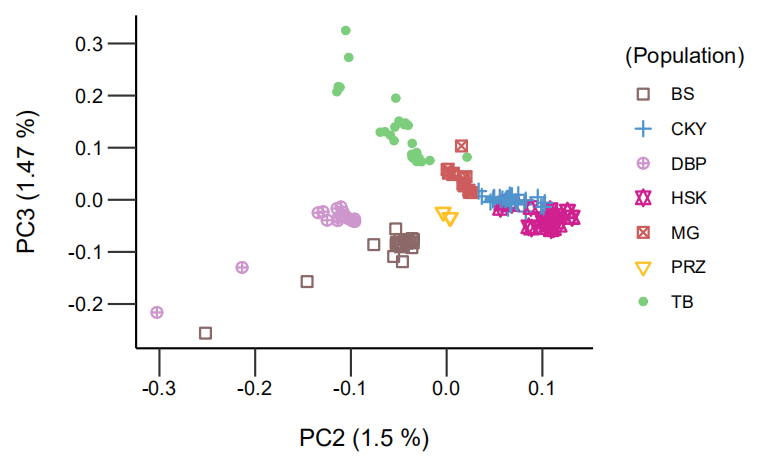


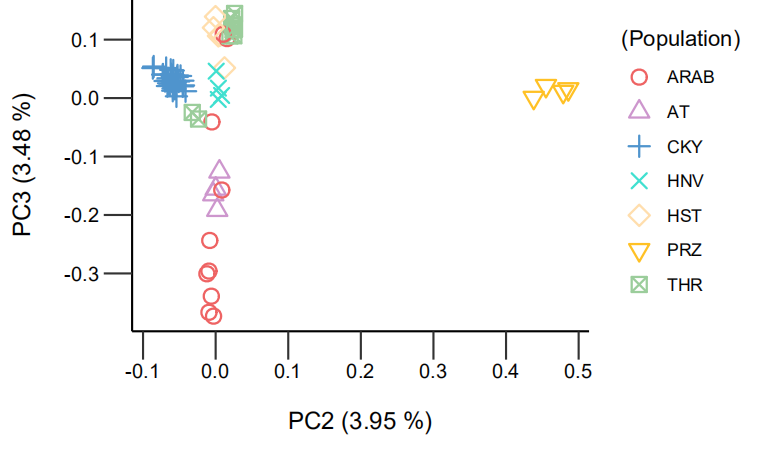


Figure S2 PCA results of the Chakouyi horse and CHBs (PC3)

.

Supplement: Supplementary file 1 [file genes-13-02411-s001.zip › genes-2037671-supplementary-proof(2022-12-18)/Figure S2.docx]

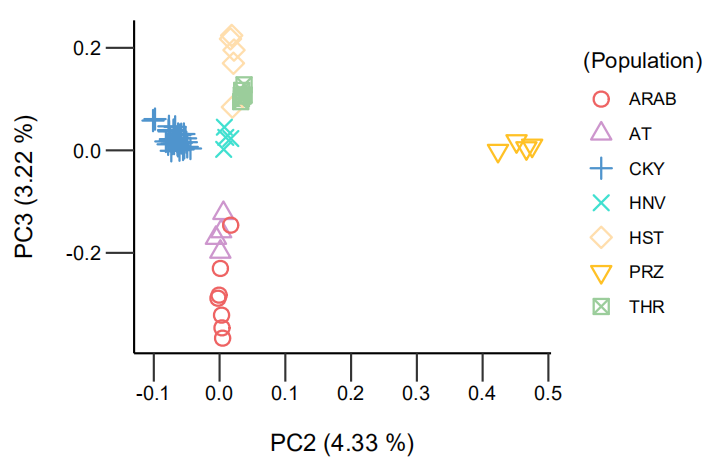

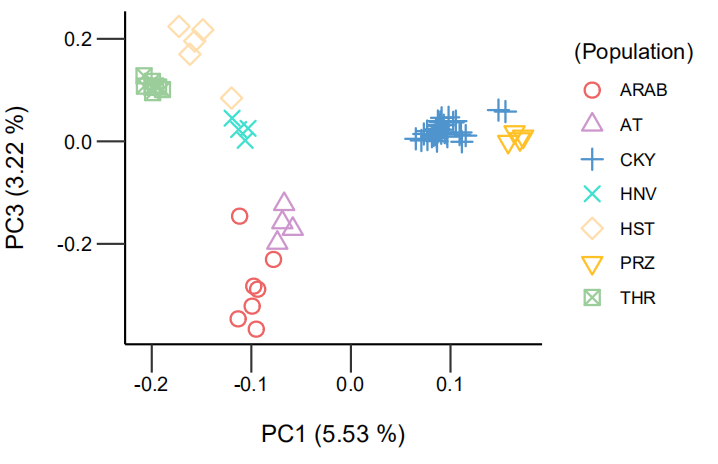


Figure S1 PCA results of the Chakouyi horse and foreign horse breeds (PC3)

.

Supplement: Supplementary file 1 [file genes-13-02411-s001.zip › genes-2037671-supplementary-proof(2022-12-18)/Figure S1.docx]
